# Learning Curve for Lymph Node Dissection Around the Recurrent Laryngeal Nerve in McKeown Minimally Invasive Esophagectomy

**DOI:** 10.3389/fonc.2021.654674

**Published:** 2021-05-20

**Authors:** Zi-Yi Zhu, Rao-Jun Luo, Zheng-Fu He, Yong Xu, Shao-Hua Xu, Qiang Zhang

**Affiliations:** Department of Thoracic Surgery, Sir Run Run Shaw Hospital, School of Medicine, Zhejiang University, Hangzhou, China

**Keywords:** hoarseness, the cumulative sum analysis, learning curve, lymph node dissection around the recurrent laryngeal nerve, minimally invasive esophagectomy

## Abstract

**Background:**

Compared to open esophagectomy (OE), minimally invasive esophagectomy (MIE) is associated with lower morbidity and mortality. However, lymph node (LN) dissection around the recurrent laryngeal nerve (RLN) is still an important factor that affects the length of the learning curve of MIE. This study aims to evaluate the surgical outcomes of the first nearly 5-year period and explore the learning curve for LN dissection around the RLN in McKeown MIE by a new single surgical team.

**Methods:**

A total of 285 consecutive patients who underwent McKeown MIE between March 2016 and September 2020 were included at our institution. According to the cumulative sum (CUSUM) analysis of LN dissection around the RLN, the patients were divided into three groups: exploration period, adjustment period, and stable period. We assessed the impact of surgical proficiency on postoperative outcomes and explored the learning curve for LN dissection around the RLN in McKeown MIE.

**Results:**

The CUSUM graph showed that a point of upward inflection for LN dissection around the RLN was observed in 151 cases. After 151 cases, LNs around the right and left RLNs were dissected thoroughly compared to the exploration and adjustment period (P = 0.010 and P = 0.012, respectively), and the postoperative incidence of hoarseness significantly decreased from 11.1 to 1.5% (P<0.001).

**Conclusions:**

Our study results revealed that not only are the LN, around the RLN, sufficiently dissected but also the incidence of hoarseness significantly decreased in the stable phase. Consequently, the learning curve length was approximately 151 cases for LN dissection around the RLN in McKeown MIE.

## Introduction

Esophageal cancer is considered the seventh most common cancer type and the sixth leading cause of cancer-related death worldwide (1, 2). More than half of esophageal cancer cases occur in China; the incidence of esophageal cancer in China ranks the sixth in malignant tumors, and the mortality rate ranks the fourth (3, 4). For patients with esophageal cancer, esophagectomy with extended lymphadenectomy remains the mainstream of multidisciplinary treatment (5). Esophageal cancer manifests as bidirectional, skipping metastasis through lymph nodes (LNs), which seriously affects the patients’ prognosis (6). LN dissection is an important part of radical resection, especially the LN around the recurrent laryngeal nerve (RLN), which leads to a high metastasis rate of esophageal cancer. Dissecting the LN around the RLN not only improves the radicality of surgical therapy but also provides adequate LN staging (7).

It is not only difficult to accomplish clearance of LN around the RLN but also easy to injure the RLN, which can lead to hoarseness, coughing, and lung infections due to the complex anatomy and the narrow space of the upper mediastinum (8). Benefiting from the advancement of thoracoscopic technology and the right thoracic approach, the bilateral tracheal–esophageal sulcus can be exposed and RLN can be identified. While LN dissection around the RLN is still an important factor that affects the length of the learning curve of minimally invasive esophagectomy (MIE), especially the LN dissection around the left RLN (7), excessive LN dissection for long-term survival may cause damage to RLN. Therefore, determining the level of experience of a surgeon in extensive lymphadenectomy around the RLN during MIE is necessary. Besides, with the increase of surgeon experience, surgical outcomes and morbidity improve, and the balance between long-term survival and postoperative safety is worth paying attention to.

The cumulative sum (CUSUM) analysis shows the deviation of each case and well presents the continuous change trend of the parameters (9). By the CUSUM, we can continuously observe performance and identify improvement regarding a predefined level of accomplishment, and it is widely used in articles that determine the learning curve. To the best of our knowledge, there are no prior studies that have focused on the learning curve for LN dissection around the RLN in McKeown MIE (9–11). This study retrospectively analyzed the clinical data of 285 patients who were treated in our department and underwent McKeown MIE of esophageal carcinoma between March 2016 and September 2020. This study aimed to explore the learning curve for LN dissection around the RLN in McKeown MIE by the CUSUM.

## Materials and Methods

### Patients

A total of 285 consecutive patients who underwent McKeown MIE with cervical anastomosis between March 2016 and September 2020 were included at our institution. The medical records of the esophageal cancer database were reviewed retrospectively, and the clinic pathological data of eligible patients were collected. We excluded patients with a previous history of gastrointestinal or lung cancer, severe comorbidities, other organ metastases, and combined other organ resection. All cases were discussed at a multidisciplinary specialist team meeting, and all operations were performed by a single surgical team with expertise in open esophagectomy (OE). Tumors were categorized based on the seventh edition of the Union for International Cancer Control (12). According to the Clavien–Dindo classification, all postoperative complications were classified (13). Hoarseness is judged by the doctor through an auditory impression. For most patients who developed hoarseness, laryngoscopy was performed to evaluate the vocal cord mobility at the Department of Otolaryngology. The research protocol for this clinical study was approved by the local ethics committee.

### CUSUM and Learning Curve

**Figure 1:**
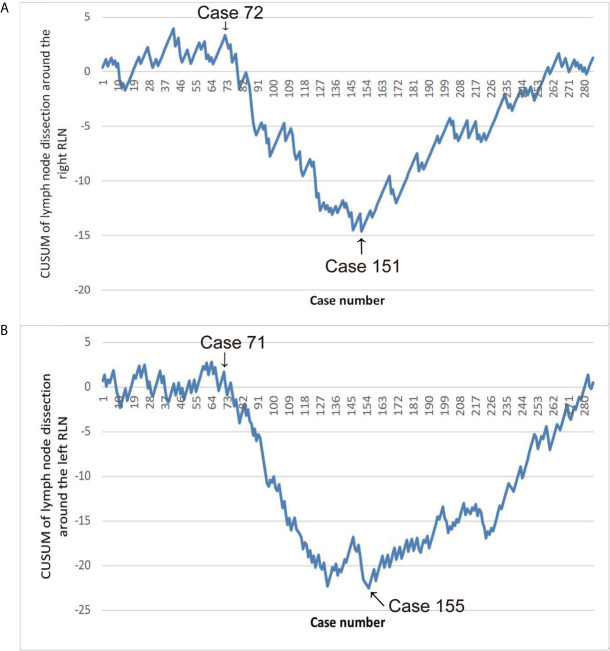
**(A)** Learning curve of lymph nodes (LN) dissection around the right RLN. **(B)** Learning curve of LN dissection around the left RLN.

The CUSUM analysis is a time-weighted control chart method which calculates the degree of deviation between the observed value of each sample and the average value and then calculates the CUSUM of deviations by summing(CUSUM = ∑outcome measure of a single case-mean outcome measure of the entire cohort). In our study, we defined CUSUM of a series of observations as $\text{SN}=\Sigma_{1}^{n}(Xi-u).$ According to the LN resection, *Xi* is positioned as three values: *Xi* = 0, which means that no LN around the RLN has been dissected; *Xi* = 1, which means that only one LN around the RLN has been dissected; *Xi* = 2, which means that more than one LN around the RLN has been dissected. *u* denotes the average value of *X* in the entire group. According to the time sequence of the operation, the patients were arranged on the horizontal axis, and based on the above formula, the CUSUM was calculated to obtain the learning curve on the vertical axis, which will show changes in performance over time. Specifically, the typical learning curve generated by the CUSUM analysis showed an initial downward slope corresponding to periods of insufficient LN dissection, and the lowest point was the cut-off point to divide the learning curve into two stages. After this upward slope was the plateau or rising slope. The learning curves of LN dissection around the right and left RLNs were made with using of CUSUM (**Figure 1**). In fact, our CUSUM learning curve showed a plateau and then showed a downward trend. After reaching the lowest point, the learning curve showed an upward slope and finally remained a plateau. We divided the learning curve into three periods according to the initial decline point and the lowest point as follows: the exploration period included cases 1–72, the adjustment period included cases 73–151, and the stable period included the final 134 cases. We collected the descriptive statistics including the patient information, tumor-related characteristics, preoperative therapy and operative outcomes. Parameters of the three groups were assessed and compared.

### Surgical Techniques

**Figure 2:**
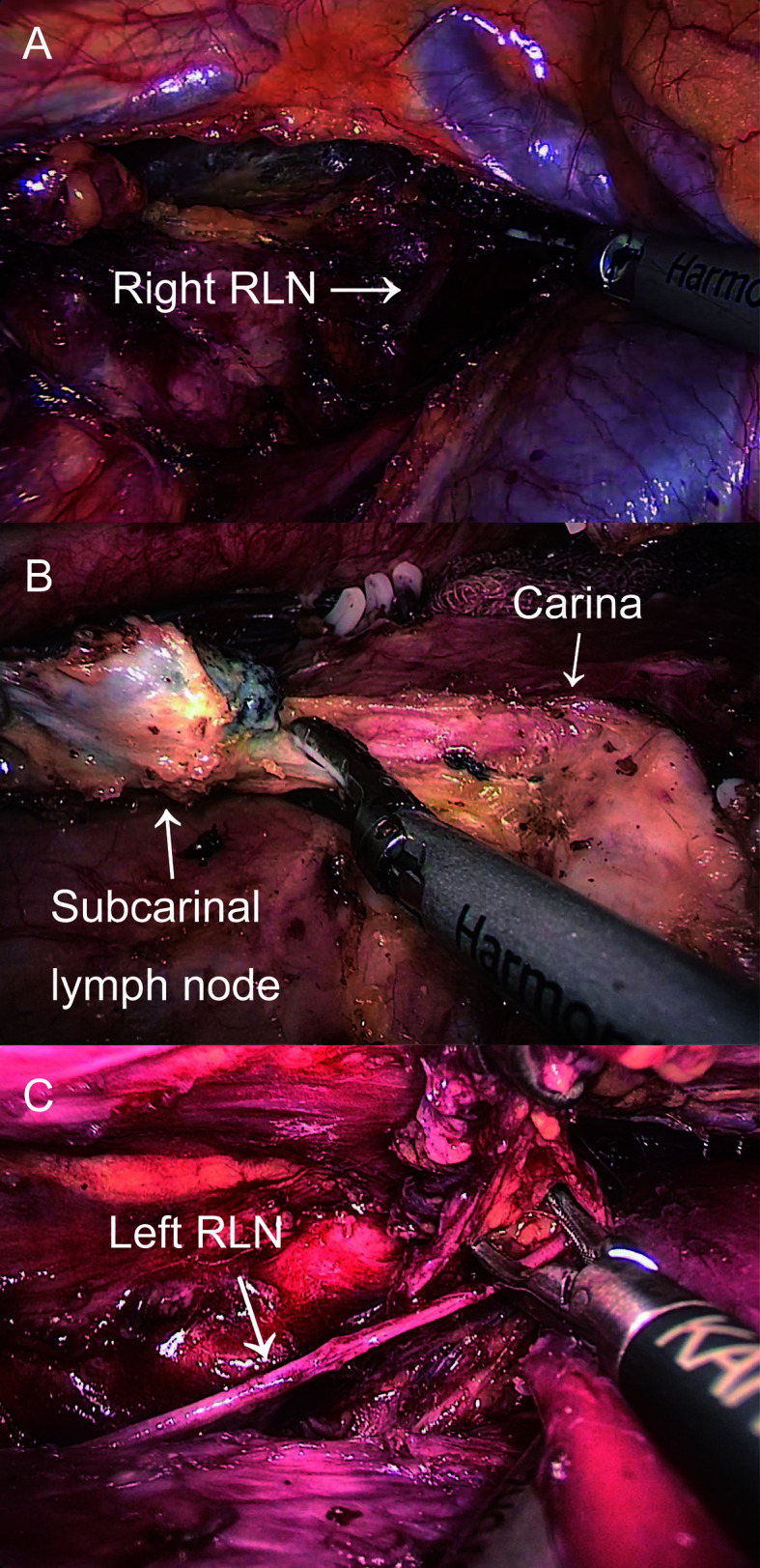
The thoracic operation. **(A)** Exposure of right RLN and LN dissection around the right RLN. **(B)** LN dissection on the carina. **(C)** Exposure of left RLN and LN dissection around the left RLN.

McKeown MIE with cervical anastomosis for esophageal cancer was implemented at our department. The details of these procedures were previously described (9). In the thoracic stage, we incised the mediastinal pleura and exposed the right RLN adjacent to the subclavian artery by following the right vagus nerve after disconnection of the azygous vein. While protecting the integrity of the nerve, LN around the RLN was dissected (**Figure 2A**). The thoracic esophagus was mobilized from azygous arch to diaphragm hiatus while protecting the thoracic duct. After dissecting the LN at the carina (**Figure 2B**), a suction device with a cotton swab was used to push the trachea forward to expose the left RLN and dissect the LN mainly by blunt separation (**Figure 2C**). In the abdominal stage, patients were turned into a lithotomy position, and the gastrocolic ligament was divided along the greater curvature with the protection of the gastroepiploic vascular arch. A tubular stomach was created outside the abdominal cavity. In the cervical stage, an oblique incision of the left neck was made, and we mobilized the cervical esophagus and cut down and pulled the gastric conduit through the esophageal bed to the left neck for gastroesophageal end-to-side anastomosis.

### Statistical Analysis

The demographic parameters of the patients were depicted using descriptive statistics. The data fitting normal distribution were expressed as mean ± standard deviation, and differences between groups were compared using Student’s *t*-test and analysis of variance with a Bonferroni multiple comparison test. Data fitting non-normal distribution were expressed as median (interquartile range), and the differences between groups were compared using the Wilcoxon rank-sum test and Kruskal–Wallis H test. Categorical variables were expressed as frequency (%) and were analyzed using the chi-square test. The statistical analysis was completed using SPSS^®^ version 20 (IBM Corp. in Armonk, NY, USA), with P <0.05 indicating a statistically significant difference.

## Results

### Demographic Parameters

A total of 285 patients underwent McKeown MIE, and no one was converted to an open operation. The average age of the 285 patients was 64.97 ± 7.19 years, and there were 253 (88.8%) males and 32 (11.2%) females. The median operative time was 240 min, and the median blood loss was 100 ml. According to our CUSUM learning curve, we divided the learning curve of LN dissection around the RLN into three periods: the exploration period included cases 1–72, the adjustment period included cases 73–151, and the stable period included the final 134 cases. **Table 1** shows the patient and tumor characteristics of three periods. No significant differences in age, gender, BMI, ASA, history of smoking, history of drinking, comorbidities, history of thoracic surgery, neoadjuvant immunotherapy, neoadjuvant radiotherapy, history of ESD, histology, and tumor location were observed, while there was a significant difference in neoadjuvant chemotherapy among the three groups. Patients who received neoadjuvant chemotherapy were more in the stable period than in the exploration period and the adjustment period.

**Table 1:** The demographics and tumor characteristics of the overall cohort.

	Exploration period (n = 72)	Adjustment period (n = 79)	Stable period (n = 134)	P
Age (y)	64.04 ± 7.20	65.92 ± 7.90	64.91 ± 6.70	0.273
Gender				0.252
Male	69(95.8)	68(86.1)	116(86.6)	
Female	3(4.2)	11(13.9)	18(13.4)	
BMI (kg/m^2^)	21.99 ± 3.16	21.96 ± 3.49	22.59 ± 3.15	0.276
History of smoking				0.11
Yes	43(59.7)	37(46.8)	60(44.8)	
No	29(40.3)	42(53.2)	74(55.2)	
History of drinking				0.403
Yes	39(54.2)	35(44.3)	61(45.5)	
No	33(45.8)	44(55.7)	73(54.5)	
ASA				0.089
II	68(94.4)	75(94.9)	133(99.3)	
I II	4(5.6)	4(5.1)	1(0.7)	
Comorbidity, n (%)				
Hypertension	21(29.2)	23(29.1)	48(35.8)	0.484
Diabetes mellitus	3(4.2)	6(7.6)	7(5.2)	0.635
Cardiovascular disease	4(5.6)	3(3.8)	9(6.7)	0.67
Obstructive lung disease	2(2.8)	5(6.3)	4(3.0)	0.406
Cerebrovascular disease	0(0)	1(1.3)	6(4.5)	0.102
History of thoracic surgery, n (%)	0(0)	1(1.3)	0(0)	0.27
Neoadjuvant chemotherapy, n (%)	3(4.2)	14(17.7)	29(21.6)	0.005
Neoadjuvant radiotherapy, n (%)	0(0)	3(3.8)	2(1.5)	0.197
Neoadjuvant immunotherapy, n (%)	0(0)	0(0)	2(0.7)	0.321
Endoscopic submucosal dissection, n (%)	0(0)	0(0)	5(3.7)	0.057
Histological type, n (%)				0.074
Squamous cell carcinoma	71(98.6)	74(93.7)	132(98.5)	
Adenocarcinoma	0(0)	1(1.3)	2(1.5)	
Others	1(1.4)	4(5.1)	0(0)	
Tumor location, n (%)				0.207
Upper	6(8.3)	4(5.1)	5(3.7)	
Middle	29(40.3)	37(46.8)	46(34.3)	
Lower	37(51.4)	38(48.1)	83(61.9)	

ASA, American Society of Anesthesiologists; BMI, body mass index.

Data are expressed as mean ± standard deviation, median (interquartile range), or number (percentage).

### Operative Outcomes and Pathological Characteristics

**Table 2** shows the operative and pathological characteristics. Significant differences were found among the three periods in operative time (P = 0.008), restore fluid diet time (P <0.001), harvested LN (P <0.001), harvested LN around the right RLN (P = 0.010), and harvested LN around the left RLN (P = 0.012), while there were no significant differences in terms of intraoperative blood loss, postoperative hospitalization days, thoracic duct ligation, and TNM stage.

**Table 2:** Operative and pathological outcomes of the three periods.

	Exploration period (n = 72)	Adjustment period (n = 79)	Stable period (n = 134)	P
Operation time (min)	245(211.25, 300)	215(205,265)	235(215, 276.25)	0.008^a,c^
Intraoperative blood loss (ml)	100(50, 100)	100(100,150)	100(50, 100)	0.481
Restore fluid diet time (d)	8(7.25, 10)	8(7, 9)	9(9, 11)	<0.001^b,c^
Postoperative hospital stay (d)	12(10, 13)	11(10, 13)	12(10, 14)	0.317
Thoracic duct ligation	27(37.5)	25(31.6)	33(24.6)	0.144
Harvested lymph nodes	33.5(25, 45.75)	24(19,32)	28(19.75, 38.25)	<0.001^a,b^
Harvested lymph nodes around the right RLN	3(1, 5)	2(1, 4)	3(2, 5)	0.010^c^
Harvested lymph nodes around the left RLN	3(0, 5.75)	1(0, 4)	2(1, 5)	0.012^a,c^
T stage				0.126
T1	20(27.8)	25(31.6)	35(26.1)	
T2	9(12.5)	12(15.2)	35(26.1)	
T3	43(59.7)	41(51.9)	64(47.8)	
T4	0(0)	1(1.3)	0(0)	
N stage				0.177
N0	30(41.7)	42(53.2)	68(50.7)	
N1	21(29.2)	24(30.4)	31(23.1)	
N2	15(20.8)	13(16.5)	25(18.7)	
N3	6(8.3)	0(0)	10(7.5)	
Pathological stage				0.127
Stage I	19(26.4)	23(29.1)	46(34.3)	
Stage II	14(19.4)	25(31.6)	25(18.7)	
Stage III	33(45.8)	30(38.0)	53 (39.6)	
Stage IV	6(8.3)	1(1.3)	10(7.5)	

Data are expressed as mean ± standard deviation, median (interquartile range), or number (percentage).

^a^indicates that there is a significant difference between the exploration period and the adjustment period. ^b^indicates that there is a significant difference between the exploration period and the stable period. ^c^indicates that there is a significant difference between the adjustment period and the stable period.

### Postoperative Complications

**Table 3** shows the details about postoperative complications. The rate of hoarseness was 11.1% in the exploration period, which was significantly higher than that in the adjustment period and the stable period (P <0.001). Among 10 patients with hoarseness in entire cohort, eight of them recovered with conservative treatment within 6 months after surgery. However, the remaining two cases in the exploration period still showed permanent hoarseness after 6 months of conservative treatment. The rate of anastomosis leakage in the entire cohort was 5.3%, and the incidence was as low as 2.2% in the stable period. In the entire group, three patients underwent secondary surgery, one underwent a debridement suture due to a severe abdominal incision infection, one with chyle leak was treated by reoperation for thoracic duct ligation, and another one was treated with surgical hemostasis due to bleeding in the thoracic cavity. Perioperative deaths occurred in two patients, one suffered from anastomosis leakage and died of cachexia 105 days after surgery, and another one suffered from severe pneumonia and died of respiratory failure 30 days after surgery. No significant differences in complications were observed among the three periods except for the hoarseness.

**Table 3:** Postoperative complications of the three periods.

	Exploration period (n = 72)	Adjustment period (n = 79)	Stable period (n = 134)	P
Anastomosis leakage	5(6.9)	7(8.9)	3(2.2)	0.086
Hoarseness	8(11.1)	0(0)	2(1.5)	<0.001^a^
Wound infection	3(4.2)	1(1.3)	6(4.5)	0.441
Pleural effusion	3(4.2)	3(3.8)	14(10.4)	0.102
Pneumonia	2(2.8)	3(3.8)	5(3.7)	0.926
Chylothorax	2(2.8)	0(0)	3(2.2)	0.362
Arrhythmia	0(0)	1(1.3)	3(2.2)	0.425
Pneumothorax	4(5.6)	0(0)	3(2.2)	0.086
ICU stay	4(5.6)	2(2.5)	4(3.0)	0.543
Secondary surgery	1(1.4)	0(0)	2(1.5)	0.558
Death	0(0)	1(1.3)	1(1.1)	0.646

Data are expressed as number (percentage).

a Statistically significant.

## Discussion

To the best of our knowledge, this is the first study on the learning curve for LN dissection around the RLN accompanied by complications in McKeown MIE. In our study, we presented our first nearly 5-year experience in McKeown MIE implemented by a new surgical team. Our study results indicated that harvested LN around both the right RLN and left RLN significantly increased in the stable period compared to the adjustment period. Besides, the incidence of hoarseness decreased significantly between the exploration period and the stable period. Consequently, improvements in precise procedure can be achieved through case accumulation and result in more LN dissections around the RLN but with a lower rate of hoarseness.

As well known, there is an association between MIE and less surgical trauma, fewer complications, lower mortality, and similar oncologic outcomes compared to OE (8, 14, 15). However, MIE is still a technically complex procedure with great challenges, especially in LN dissection around the RLN (16). Therefore, long-term training and experience are required to be familiar with the process, which is considered as a learning curve. Previous studies have focused on the learning curves of McKeown MIE, and the endpoints were mainly intraoperative and postoperative outcomes, including operative time, blood loss, harvested LN, and hospital length of stay (9, 16–22). According to research studies, based on improved outcomes of operative time, the learning curve for McKeown MIE has been a wide range of length from 20 to 175 cases. There are a few research studies on learning curves of surgical procedures which have focused on postoperative complications (9, 23), but not on the learning curve of LN dissection around the RLN and hoarseness. Most previous studies arbitrarily divided groups and defined learning curves based on the time axis, which is prone to bias because the authors can group according to their data (18–22). In a few studies, CUSUM analysis was used to determine the length of the learning curve (9, 16, 17). Additionally, a longer length of the learning curve for McKeown MIE was found in the studies that include more patients. In other words, the learning curve length found in the small sample study may be biased, because we can never be sure whether the learning curve is longer than the total small sample size in small sample research. Therefore, this study was conducted to overcome the limitations of prior studies. In this study, we retrospectively analyzed the clinical data of 285 patients operated on by the same surgical team in our department who underwent McKeown MIE of esophageal carcinoma between March 2016 and September 2020. Combined with hoarseness, the precise length of the learning curve for LN dissection around the RLN in McKeown MIE was identified by performing the CUSUM analysis.

Early in the research, the learning curve of LN dissection around the right and left RLN separately was identified using CUSUM analysis. Since hoarseness can be caused by injury of either side of RLN, analyzing the complications of hoarseness independently in the learning curve is impossible. Secondly, in this study, the learning curve of LN dissection around the right and left RLN was the same with plateau, descending, and ascending periods. Third, the entire group was divided into three groups separately according to the two learning curves as criteria for grouping. Corresponding to the two grouping methods, the sample sizes are similar. This study adopted the learning curve of LN dissection around the right RLN as the basis for grouping based on the above reasons. The learning curve was divided into three periods: the exploration period included cases 1–72, the adjustment period included cases 73–151, and the stable period included the final 134 cases.

With the accumulation of experience in McKeown MIE, the improvement of perioperative parameters has become obvious. The learning curve of dissection LN around the RLN consisted of three components: thorough LN dissection accompanied by a high incidence of hoarseness (exploratory period), reduced dissection to reduce the rate of hoarseness (adjustment period), and increased number of nodules around the RLN with a low incidence of hoarseness (stable period). These aspects corresponded to improvements in anatomical dissection, functional preservation, and oncological treatment. The above steps are not independent but interrelated. It is not easy for beginners to dissect the LN around the RLN due to the complex anatomy and the narrow space of the upper mediastinum. During the exploratory period, our team tried LN dissection around the RLN, which was accompanied by an 11.1% incidence of hoarseness. We appropriately reduced lymphatic dissection around the RLN to reduce the incidence of hoarseness, and no hoarseness occurred during the adjustment period. Regarding the improvement of the adjustment period, there was a conflict between oncological treatment and functional preservation during surgery. With the accumulation of experience during periods, some improvements were implemented to achieve LN dissection around the RLN thoroughly with a low incidence of hoarseness, including full exposure of the tracheal–esophageal sulcus, extensive use of blunt separation, and less use of energy devices. Additionally, improvement in precise operation leads to refinement, achieved through the accumulation of cases, and results in the complementarity of oncological treatment and functional preservation. Our study results indicated that not only are the LN around the RLN sufficiently dissected but also the incidence of hoarseness significantly decreased in the stable phase. Consequently, the learning curve length was approximately 151 cases for LN dissection around the RLN.

This study showed that more patients underwent McKeown MIE after neoadjuvant chemotherapy in the adjustment period and stable period. Although neoadjuvant chemotherapy can reduce tumor staging and improve survival rate, it can also cause necrosis and fibrosis, especially around the tumor, which complicates the surgical process (24, 25). Our results revealed that the surgical proficiency could overcome the increase in the difficult cases. The factors influencing the implementation of LN dissection around the RLN during McKeown MIE, in terms of lower incidence of hoarseness, are multifactorial. In addition to the technical capabilities of the surgeon team, the excellent cooperation and support from different team members, including anesthesiologists, nurses, and rehabilitation trainers, were the more important factors contributing to the learning curve for McKeown MIE.

There are some limitations to this study. First, the nearly 5-year experience of a single high-volume center was presented; this study was a retrospective cohort study in a single institution. Second, the difference in the experience levels of the surgeons will contribute to different learning curve lengths in similar operations. Thus, to address this, the data of multiple centers will be analyzed to determine the average length of the learning curve in the future.

In conclusion, proficiency in precise procedure can be achieved through case accumulation and result in more LN dissections around the RLN but with a lower rate of hoarseness. LN dissections around the RLN in McKeown MIE could be performed proficiently and safely after approximately 151 cases in one surgical group.

### Equations

$$\text{SN}=\Sigma_{1}^{n}(Xi-u).$$

According to the LN resection, *Xi* is positioned as three values: *Xi* = 0, which means that no LN around the RLN has been dissected; *Xi* = 1, which means that only one LN around the RLN has been dissected; *Xi* = 2, which means that more than one LN around the RLN has been dissected. *u* denotes the average value of *X* in the entire group.

## Data Availability Statement

The original contributions presented in the study are included in the article/supplementary material. Further inquiries can be directed to the corresponding author.

## Ethics Statement

The studies involving human participants were reviewed and approved by the ethics committee. Written informed consent for participation was not required for this study in accordance with the national legislation and the institutional requirements.

## Author Contributions

Collection of data: R-JL, Z-YZ, Z-FH, YX, S-HX, and QZ. Analysis of data: R-JL, Z-YZ, and QZ. Writing of this paper: R-JL and Z-YZ. General supervision of the research group: R-JL and Z-YZ. All authors contributed to the article and approved the submitted version.

## Conflict of Interest

The authors declare that the research was conducted in the absence of any commercial or financial relationships that could be construed as a potential conflict of interest.
